# A simple and low impact glove tip to achieve colonic endoscopic submucosal dissection with adaptive traction in patients with stoma

**DOI:** 10.1055/a-2301-8107

**Published:** 2024-04-29

**Authors:** Elena De Cristofaro, Pierre Lafeuille, Jérôme Rivory, Rosario DʼAlmeida, Louis Jean Masgnaux, Alexandru Lupu, Mathieu Pioche

**Affiliations:** 1 9318 Department of Systems Medicine, Gastroenterology Unit, University of Rome Tor Vergata, Rome, Italy; 2 Gastroenterology and Endoscopy Unit, Edouard Herriot Hospital, Hospices Civils de Lyon, Lyon, France

Colonoscopy in patients with a stoma is burdened by some technical challenges, such as the difficulty in inflating the colon, which collapses because of gas leakage through colostomy. This makes certain endoscopic procedures, like endoscopic submucosal dissection (ESD), particularly challenging and time-consuming. The use of devices, like an overtube with a valve, can be useful because it helps maintain insufflation through its unique insufflation cap. However, this device is not always available and does not have a negligible ecological and economic impact.

For this reason, we have developed a simple and low impact system to avoid gas leakage through a stoma: a glove fixed around the stoma trapping the air inside ( Fig. 1 ).

**Fig. 1:**
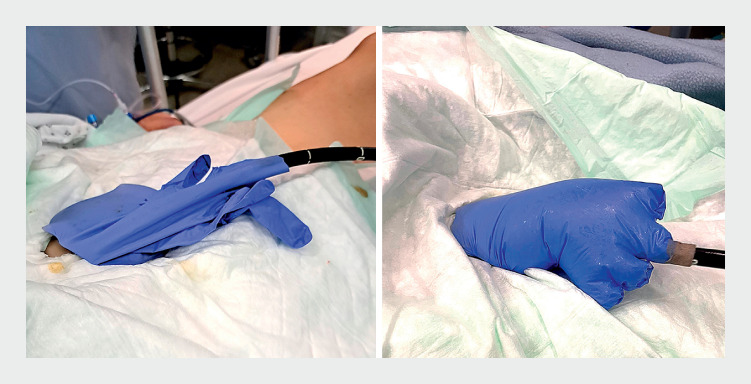
Glove fixed around the stoma before (on the left) and after (on the right) colonic insufflation.

Herein, we report two cases of patients with a sigmoid stoma for distal cancer who also had a second superficial lesion in the colon, justifying a quick R0 endoscopic resection to obtain histology before management of the more advanced distal lesion. Those colonic lesions were resected successfully with ESD using an adaptative traction device (A-TRACT) whose benefits in ESD have already been reported 1 2 3 4 5 ( Video 1 ).

A simple and low impact glove tip achieves colonic endoscopic submucosal dissection with adaptive traction in patients with a stoma.Video 1

The first case is an 83-year-old patient with a large (60 mm), granular laterally spreading tumor granular (LST) with a macro-nodule located in the right flexure. The second is a 73-year-old patient who was referred for resection of a homogeneous granular LST (20 mm) at the level of the ileocecal valve. After circumferential incision and trimming, adaptive multipolar traction was applied allowing the start of dissection with good submucosal exposure. The traction devices were tightened after cutting half of the lesions to re-establish proper traction. The procedures ended without adverse events and with a good insufflation during the procedures.

We can hypothesize that such a simple and low impact system, combined with an adaptive traction strategy, could facilitate the intervention for complex lesions in patients with a stoma.

Endoscopy_UCTN_Code_TTT_1AO_2AC

## Notes

Endoscopy E-Videos https://eref.thieme.de/e-videos
*E-Videos* is an open access online section of the journal *Endoscopy* , reporting on interesting cases and new techniques in gastroenterological endoscopy. All papers include a high-quality video and are published with a Creative Commons CC-BY license. Endoscopy E-Videos qualify for HINARI discounts and waivers and eligibility is automatically checked during the submission process. We grant 100% waivers to articles whose corresponding authors are based in Group A countries and 50% waivers to those who are based in Group B countries as classified by Research4Life (see: https://www.research4life.org/access/eligibility/ ). This section has its own submission website at https://mc.manuscriptcentral.com/e-videos .
